# Fever of Unknown Origin due to Primary Hepatic Diffuse Large B-cell Lymphoma: A Case Report

**DOI:** 10.7759/cureus.4220

**Published:** 2019-03-11

**Authors:** Fady Farag, Rewais Morcus, Preethi Ramachandran, Urvishkumar Rajivkumar Pasrija, Jen Chin Wang

**Affiliations:** 1 Internal Medicine, Brookdale University Hospital and Medical Center, Brooklyn, USA; 2 Oncology, Brookdale University Hospital and Medical Center, Brooklyn, USA

**Keywords:** diffuse large b cell lymphoma, primary hepatic lymphoma, nhl, cd5 positive, fever of unknown origin

## Abstract

We present a rare case of primary hepatic lymphoma. An 82-year-old female patient presented with altered mental status, and fever. Her labs were significant for abnormal liver functions with markedly elevated lactate dehydrogenase. All infectious and auto-immune workup was negative. Imaging studies were only significant for hepatosplenomegaly, and no liver masses were detected. A liver biopsy was diagnostic of CD5+ CD20+ diffuse large b-cell lymphoma of the liver. Chemotherapy was planned with rituximab combined with cyclophosphamide, doxorubicin, vincristine, and prednisone (R-CHOP). Unfortunately, the patient died before initiation of therapy. This case would highlight the importance of early liver biopsy in patients with abnormal liver functions and with no clear explanation, even if there were no discrete masses on computed tomography (CT) or magnetic resonance imaging (MRI). Lymphomas and other infiltrative processes should be considered in the differential diagnosis in such cases.

## Introduction

A primary hepatic lymphoma (PHL) is an extra-nodal form of non-Hodgkin’s lymphoma (NHL). These are rare malignancies with overall poor prognosis. The prevalence of primary hepatic lymphoma is 0.4% of all extra-nodal NHL. This type of lymphoma usually poses a challenge in diagnosis, giving its rarity, and non-specific finding, whether laboratory or imaging studies. In this case, we present a patient who came with mental status changes and fever and was diagnosed with CD5 + diffuse large B-cell lymphoma (DLBCL) of the liver after extensive workup.

## Case presentation

**Figure 1:**
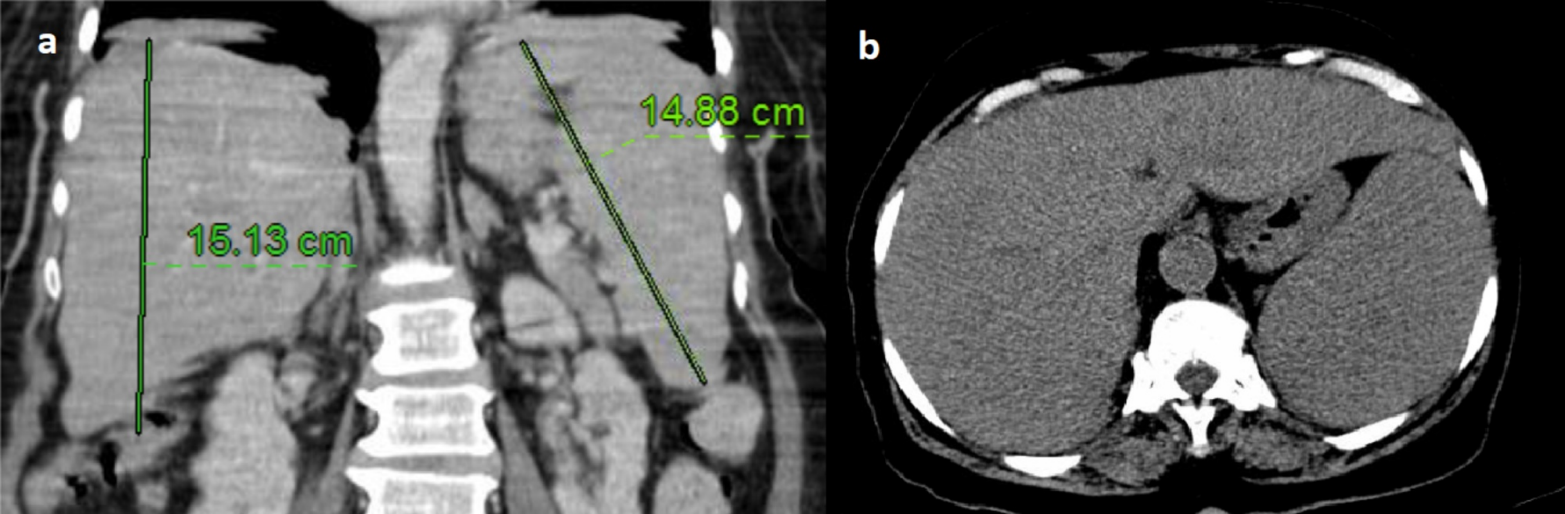
Computed tomography (CT) abdomen with contrast. (a) Coronal section showing hepatosplenomegaly. (b) Axial section showing no discrete masses.

We report a case of primary liver lymphoma with a rare immunophenotypic subtype and an unusual presentation. An 82-year-old female was found less responsive by her daughter. Prior to this event, she was complaining of generalized weakness, nausea, and abdominal pain. Emergency medical services (EMS) reported that the patient was hypotensive with systolic blood pressure in the 90s mmHg. On arrival, her vitals were significant for the temperature of 39.2 Celsius, heart rate of 112 beats/min, respiratory rate of 20/min, blood pressure of 112/47 mmHg, and oxygen saturation of 92% on room air. Initial labs revealed hemoglobin of 11.2 g/dl, platelets (PLT) of 189, white blood cell count (WBC) of 4.6, alanine aminotransferase (ALT) of 63, aspartate aminotransferase (AST) 182, alkaline phosphatase (ALP) of 242, gamma-glutamyl transferase (GGT) of 252, total bilirubin of 1.2, direct bilirubin of 0.8, albumin of 2.5, triglycerides of 321, total cholesterol of 107, low-density lipoprotein (LDL) 22, and high-density lipoproteins (HDL) 2. Lactate dehydrogenase (LDH) was elevated to 4300. Urinalysis was negative for infection. Chest X-ray showed bilateral perihilar reticulonodular opacities, and no definite infiltrates. CT abdomen and pelvis showed periportal lucency, trace ascites and hepatosplenomegaly (Figure 1).

Initial impression was sepsis secondary to an unknown source. Blood cultures were taken, and she received broad-spectrum antibiotics and fluids according to the sepsis protocol. Ultrasound of the abdomen showed cholelithiasis with no obstruction, enlarged echogenic liver, and splenomegaly.

**Figure 2:**
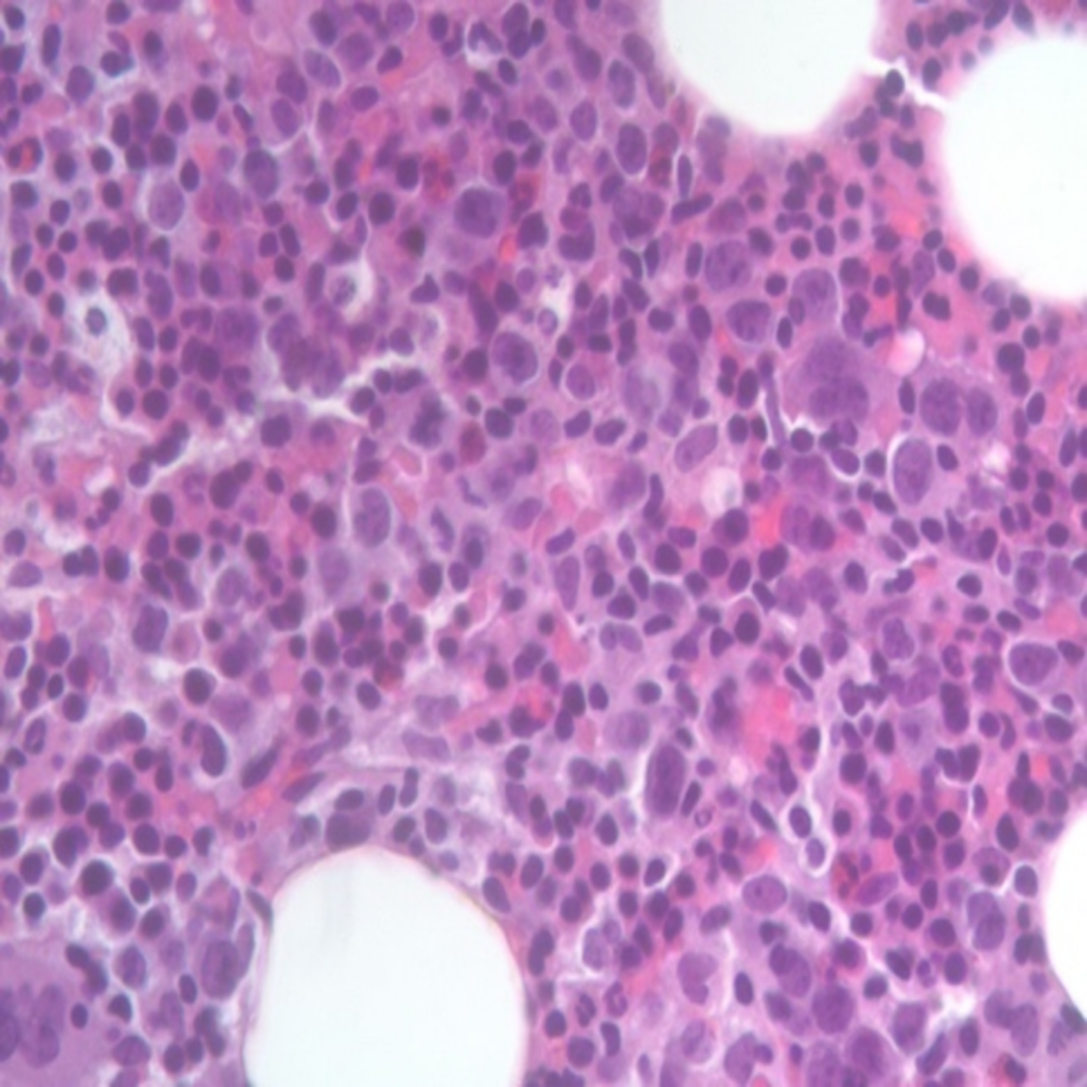
Hypercellular bone marrow with no dysplastic cells.

In the following days, she became more lethargic with waxing and waning mental status. She had daily spikes of fever despite antibiotics then she developed pancytopenia. Lactate remained elevated despite fluid resuscitation. CT chest was done in an attempt to localize the source of infection, but it failed to show any evidence of it. Lumbar puncture was negative for acute infections. Acute cholecystitis was suspected but was ruled out with hepatobiliary iminodiacetic acid (HIDA) scan. Acute viral hepatitis, tuberculosis, syphilis, human immunodeficiency virus (HIV), herpes simplex virus (HSV), Epstein-Barr virus (EBV), auto-immune hepatitis, malaria were all ruled out. Peripheral smear showed some target cells, stomatocytes, and occasional schistocytes, reduced number of WBC and PLT with no dysplastic cells or blasts. Given the persisting fever, pancytopenia, splenomegaly and elevated TG levels, hemophagocytic lymphohistiocytosis (HLH) was suspected. Another differential at this point was myelodysplastic syndrome, other hematological disorder, or another connective tissue disease. Bone marrow biopsy was done, and the smear showed hypercellular marrow for age, myeloid to erythroid ratio was 3:1, cells with a full spectrum of maturation and no dysplastic cells (Figure 2).

Flow cytometry analysis showed a small CD5+ monoclonal B-cell population (1% of cellularity) with no evidence for abnormal myeloid maturation or an increased blast population. The significance of this B-cell population was undetermined as it was quite small. Fluorescence in situ hybridization (FISH) studies revealed no evidence of deletion of 5q or monosomy 5, no evidence of monosomy 7 or deletion of 7q, no evidence of trisomy 8 (+8), no evidence of deletion of 20q12, no evidence of CCND1-IGH [translocation t(11;14)] gene rearrangement, and no evidence for trisomy 11 or gain of 11q, no evidence of p53 (17p13) deletion or amplification, and no evidence of BCR/ABL rearrangement. No detected genomic alterations were identified in the sequencing study.

**Figure 3:**
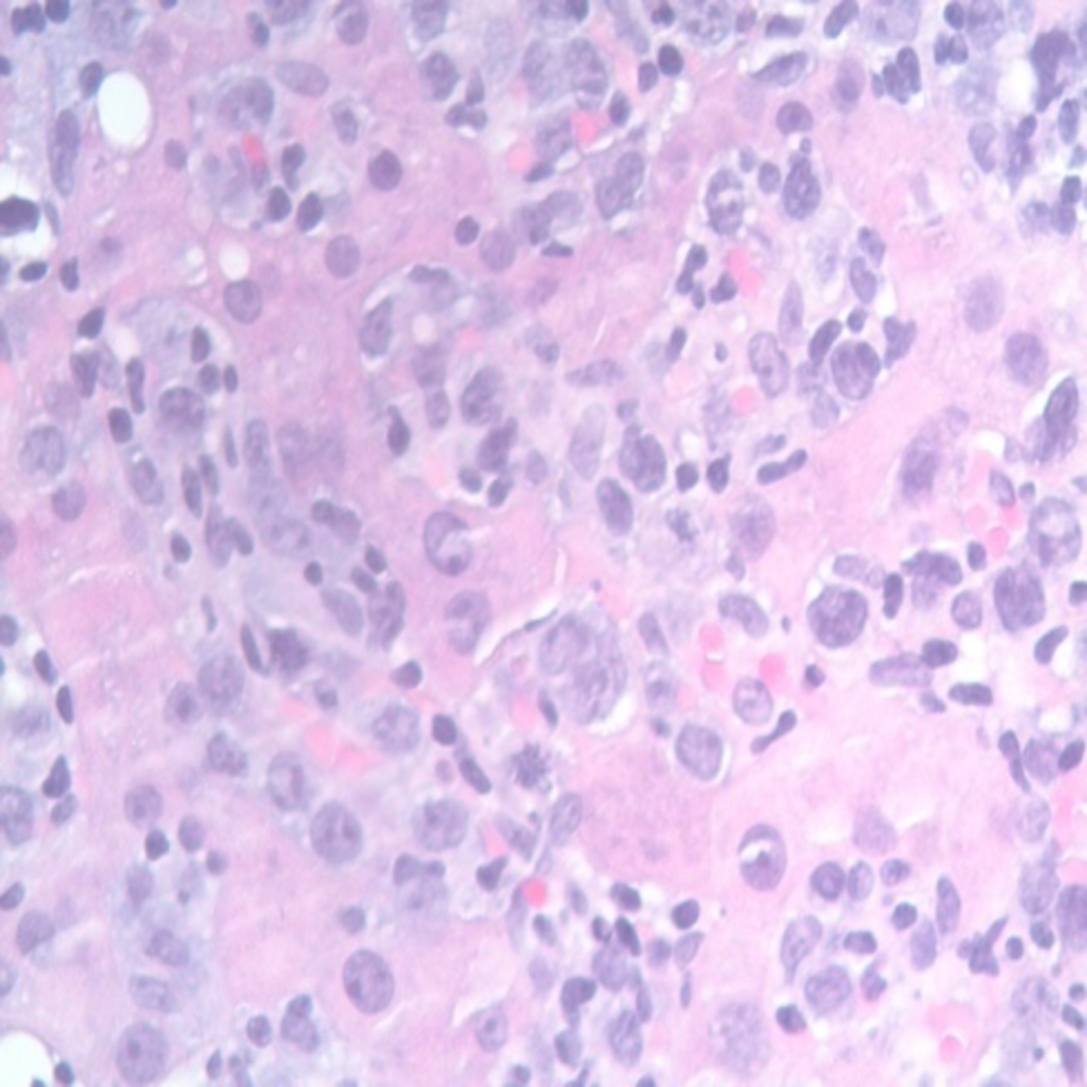
Liver biopsy stained with haematoxylin and eosin (H&E) stain showing large abnormal lymphoid cells with abnormal mitosis.

Pending the complete report of the bone marrow and cytogenetics (which came back some days later), a trail of steroids with dexamethasone for the treatment of HLH was given. CD25 (IL-2) was checked to confirm the diagnosis. After steroids, her mental status started to improve for the first time since admission. However, CD25 levels came back low, making the diagnosis of HLH less likely. Liver biopsy revealed a prominent infiltrate of large pleomorphic lymphoid cells distributed in a sinusoidal pattern (Figure 3).

**Figure 4:**
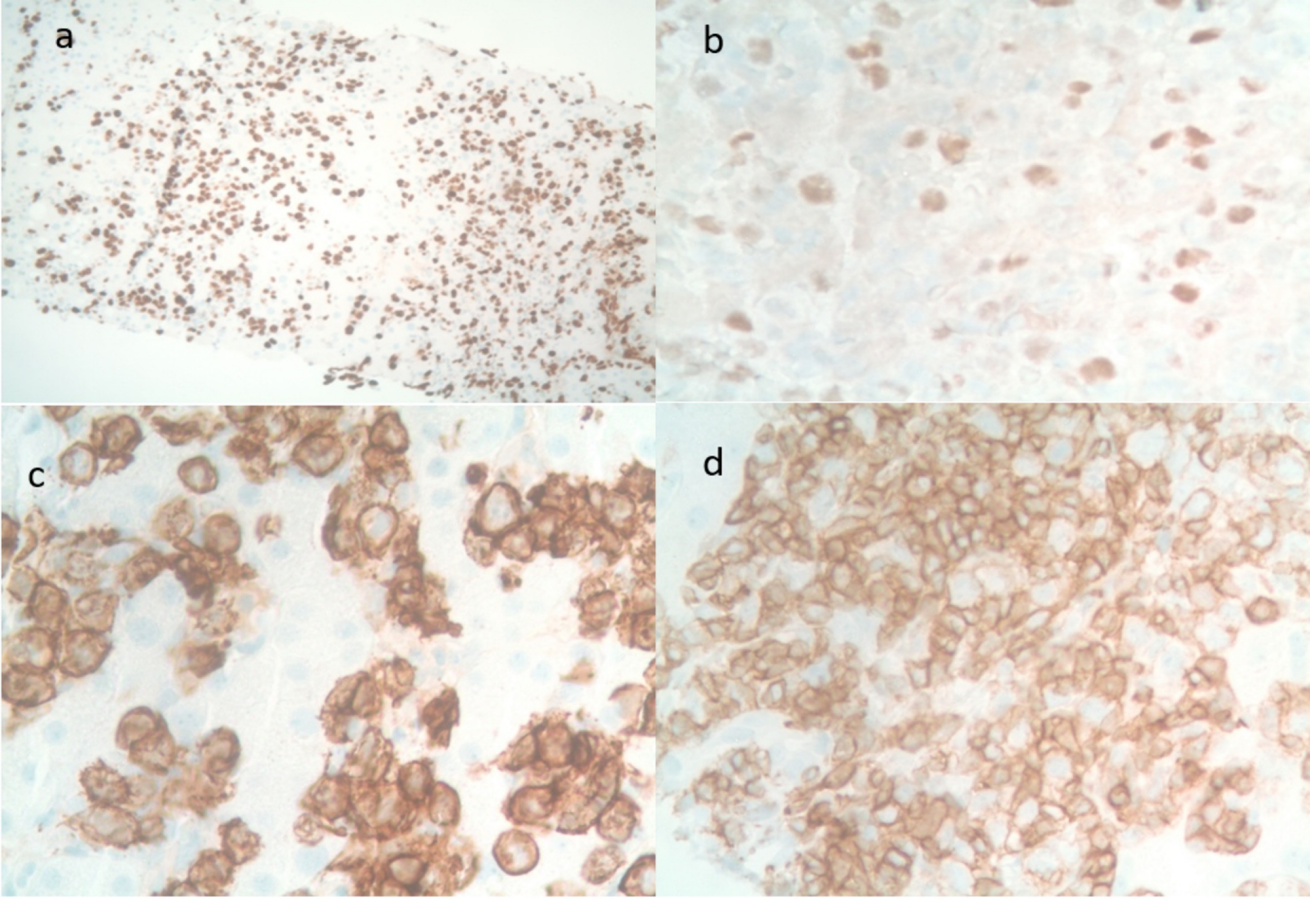
Sections of the liver with immunohistochemical staining. (a) Ki67 80% positive, (b) lymphoid cells positive for BCL6, (c) lymphoid cells positive for CD20 positive, (d) lymphoid cells positive for CD5 positive.

These cells exhibited the following phenotypes: CD20+, MUM-1+, CD5+, CD10-, cyclin-D1-, BCL-2+, BCL-6+, CD30-, c-MYC-, Ki-67 (80%) (Figure 4).

These findings supported a diagnosis of CD5 positive diffuse large B-cell lymphoma that can further be classified as diffuse large B-cell lymphoma non-germinal center type. Given the findings in the liver, the marrow findings were characterized as marrow involvement by B-cell non-Hodgkin lymphoma. A decision was made to start a reduced dose of intravenous rituximab combined with cyclophosphamide, doxorubicin, vincristine, and prednisone (R-CHOP). However, the patient deteriorated rapidly. She was intubated for hypoxic respiratory failure and died on day 21, before chemotherapy, after a prolonged complicated hospital course.

## Discussion

PHL is a form of NHL with the primary site in the liver [1, 2]. DLBCL constitutes about 40% of non-Hodgkin’s lymphomas [3]. De novo CD5+ DLBCL is a subtype which comprises only 5-10% of DLBCL [4]. In CD5+ DLBCL, female moderately predominates with a ratio of 1.2:1. The mean age is 70 years, while in CD5-negative DLBCL, males prevail with a ratio of 1.4:1. Most patients with CD5-positive DLBCL have a high International Prognostic Index (IPI) score [5, 6]. Clinically, CD5 expression in diffuse large B cell lymphoma is associated with an aggressive course [7]. CD5 adds to B-cell survival advantage through stimulation of autocrine IL-10 [8]. Presentation with elevated LDH, B symptoms, extranodal involvement, poor performance status, and more frequent central nervous system (CNS) involvement is seen more with CD5 expression [9]. Rituximab-based chemotherapy has improved the overall survival (OS) in CD5+ DLBCL, but the OS remains much lower compared to CD5- DLBCL [10, 11]. To be considered a primary hepatic lymphoma, the liver has to be the only or the major organ affected with lymphoma with only minimal extra-hepatic involvement [12]. PHL can present as a single hepatic mass or multiple nodules. Less commonly, it presents with no discrete liver masses [13]. This makes the diagnosis very challenging, as it was in our case. Some factors are associated with PHL namely: HIV, EBV, hepatitis B virus (HBV), hepatitis C virus (HCV), or any other immunocompromised state [14]. Different modalities for treatment of PHL were suggested, including chemotherapy, radiotherapy, and surgery. However, prognosis remains very poor with a median survival of 33 weeks [15]. Our patient did not have any of the mentioned risk factors, nor was she immunocompromised. And the imaging studies were unrevealing. This case was a diagnostic challenge by presenting as a rare and unique immunotypic subtype of DLBCL in a unique location with poor prognostic features. We wanted to illustrate the importance of early liver biopsy in the cases with unexplained elevated liver enzymes and LDH. In such cases, infiltrative processes including lymphoma must be ruled out.

## Conclusions

We present a rare case of primary hepatic lymphoma. Our case is unique because of the rarity of this tumor and non-specific presentation. This posed a diagnostic challenge. There was diffuse involvement of the liver with no discrete masses on imaging studies. It is very important to consider this disease and other infiltrative processes in the differential diagnosis of abnormal liver function tests with unclear etiology. In these cases, early liver biopsy should be pursued. The overall prognosis of primary hepatic lymphomas remains, unfortunately, poor. Early recognition of this disease might improve the prognosis.
